# Seroprevalence Study of Leptospirosis Among Rice Farmers in Khuzestan Province, South West Iran, 2012

**DOI:** 10.5812/jjm.11536

**Published:** 2014-07-01

**Authors:** Seyed Mohammad Alavi, Mohammad Mehdi Khoshkho

**Affiliations:** 1 Health Institute, Jundishapur Infectious and Tropical Diseases Research Center, Jundishapur University of Medical Sciences, Ahvaz, IR Iran; 2 Infectious Diseases Department , Razi Hospital, Medical College, Jundishapur University of Medical Sciences, Ahvaz, IR Iran

**Keywords:** Occupational Diseases Leptospirosis, IgM-ELISA, Serology, Iran

## Abstract

**Background:**

Leptospirosis as an emerging infectious disease is considered as an important public health problem worldwide.

**Objectives:**

The current study aimed to identify potential risks for leptospirosis among rice farmers in Khuzestan province, Iran.

**Patients and Methods:**

A cross-sectional study was carried out in Khuzestan, South west Iran, from October to December, 2012. Randomly selected participants were placed in two groups: Rice farmers as cases, and non-farmers as controls. Blood samples obtained from the participants were tested for IgM anti-leptospira antibodies using Serion ELISA classic ESR 125M. The assays were performed and interpreted according to the manufacturer’s instructions. A questionnaire including variables related to *Leptospira* spp. exposure was administered to each participant. SPSS software version 16 was employed; Chi square and Fisher’s exact tests were used to analyze data.

**Results:**

Of the total 288 samples, 65 (22.5%) were positive for IgM anti-*leptospira* antibodies. Among the positive samples, 52 (36.1%) were from the case (rice farmer) and 14 (9.7%) from the control groups. There was a significant difference between the case and control groups regarding *leptospiral* infection (P < 0.0001). Mean age in male and female in the case and control groups were 44.2, 41.9 and 43.5, 41.2 years, respectively (P > 0.05). In the case group, males and those more than 35 years were at a higher risk of infection.

**Conclusions:**

Rice farmers are significantly infected with leptospirosis, and infection prevalence is highly affected by gender (male) and age (above 35 years). Rice farming parts of Khuzestan, Iran may be considered as endemic for leptospirosis.

## 1. Background

Leptospirosis, as a zoonotic infectious disease, is frequently found in undeveloped areas. Many wild and domestic animals are the main reservoirs for the *Leptospira* species. Transmission usually results from direct or indirect exposure to the urine or other tissues of the infected animals (1). Indirect exposure, which is more common, occurs via contact with contaminated water and wet soil, when *Leptospira* species enters the body through abrasions in skin, intact skin, mucosa such as respiratory tract and conjunctiva. Early symptoms of leptospirosis are usually undiagnosed clinically (2). Early diagnosis is essential, since the untreated illness can progress rapidly and mortality rates are high in severe cases (3).

Leptospirosis outbreaks have been reported following swimming in and drinking contaminated water (4). Certain occupational groups, including rice farmers, fishermen, sugarcane workers, sewer workers, and military personnel, are considered to be at increased risk of leptospirosis (5). In the recent decades, leptospirosis as an emerging infectious disease has been considered as an important public health problem worldwide (6). It occurs in urban environments, as well as rural regions. Mortality has remained significant, because of delays in diagnosis and adequate clinical suspicion (7). Human leptospirosis has been reported in Northern area of Iran; Guilan and Mazindaran (8, 9). Spread of animal leptospirosis is widely observed in most areas of Iran (9, 10).

Diagnosis is definitely made by blood or urine culture, but it takes time and the growth is unreliable, therefore diagnosis usually depends on serologic tests in the presence of clinical assessment and history of exposure to leptospiral sources. The definitive serologic test is the microscopic agglutination test (MAT) in which live antigen suspensions are titrated with patients’ sera and then inspected microscopically for agglutination (3, 11). Other serological diagnostic tests with similar sensitivity are used to identify the recent or current infection (12). Cross reaction IgM antibody may be associated with other spirochete organisms and autoimmune diseases (3). Although clinical spectrum of leptospirosis is broad, ranging from subclinical to severe fatal illness, the most frequent initial clinical presentation is fever, head ache, and myalgia (3).

Every year, thousands of people in cities of Iran travel to villages throughout the country. This travel has led to an increasing risk for contact with pathogens through exposures to lakes, rivers, farms, and contact with animals. This event is sometimes results in the illnesses which may be unfamiliar to practitioners in the health network setting, and symptoms may remain unrecognized (13). Prevention of human leptospirosis relies on exposure avoidance, benefits of pre-exposure chemoprophylaxis is in doubt (14, 15). The disease is usually underreported, but recent surveillance attempts suggested that it is the most common zoonotic infection worldwide (3). There are few and limited reports of leptospirosis in agricultural and fishery areas of Iran (10, 16). 

## 2. Objectives

The current study aimed to identify potential risks for leptospirosis among rice farmers in Khuzestan Province, Southwestern, Iran, which is suspected to have latent epidemic of leptospirosis.

## 3. Patients and Methods

### 3.1. Study Design and Date

A cross-sectional study was carried out in the rural areas of Khuzestan province, Southwestern Iran, from October to December, 2012.

### 3.2. Place, Population, and Data Collection

Khuzestan has a population around 4,800,000. Farming is the primary occupation in the rural community with especial interest to rice farming in some areas. Rural households in rice farming areas (Shadegan, Baghemalek and Ahvaz) were randomly selected for this study using detailed maps of the cities in Health Network setting. A standardized questionnaire that included variables related to *Leptospira* species exposure was administered to each participant. Required data collected by this questionnaire included socioeconomic status, sanitation system, water source, food source, animals, and rats or rodents contact. In addition, demographic information, occupational and environmental exposures were included.

### 3.3. Definitions and Sample Size

Participants were placed in two groups: Rice farmers as the case group, and the other people not engaged in farming as the control group. Sample size for case group was calculated as 144, according to statistics formula. For each case a person was randomly selected (not engaged with rice farming) from the people in the same village as control. Considering the possibility that some villagers may not participate in the study, about 165 samples were taken for the cases and the same for the control groups. This project was reviewed and approved by Research Deputy and Ethics Committee of Medical College and Infectious and Tropical Diseases Research Center of Jundishapur University of Medical Sciences.

### 3.4. Laboratory

Blood samples were collected by the trained lab personnel during the interview and sent to the medical laboratory for serological examination. Collected blood specimens were frozen at -20 ºC until testing. The samples were tested for IgM anti-leptospira antibodies using an IgM anti-leptospiral enzyme-linked immunosorbent assay using Serion ELISA classic ESR 125M kits (D-97076 Wurzburg, Germany). The assays were performed and interpreted according to the manufacturer’s instructions. To standardize the tests, first all associated IgM antibodies against other spirochetes as well as rheumatoid factors (RF) were removed with the laboratory methods, and then IgM anti-leptospira –antibody was measured. A test value of more than 20 IU/mg was considered positive and interpreted as an evidence of the recent or current infection. Values 15 to 20 IU/mg considered as borderline, and lower than 15 as negative.

### 3.5. Statistical Analysis

 SPSS software version16 was employed for descriptive statistics and subsequent multivariable analyses. Chi square and Fisher’s exact tests were used to analyze data in both groups. Differences with P-values less than 0.05 were considered as significant. The 95% confidence limits for the odds ratios were calculated.

## 4. Results

Three hundred and twenty six subjects were enrolled. Thirty eight subjects declined due to fear of phlebotomy and were excluded. Blood samples were tested from the remaining subjects. Only the 288 subjects with blood test results were included in the final analysis. Of the 288 tested samples, 65 were positive for IgM anti-leptospira antibodies, indicating recent leptospiral infection (22.5%). Among the positive samples, 52 (36.1%) were from the case (rice farmer) and 14 (9.7%) from the control groups. There was a significant difference between the case and control groups regarding leptospiral infection (P < 0.0001).

The mean age of the case group was 43.5 years (range: 21-72 years) and the same for the control group was 42.4 years (range: 14-82 years). Mean age in male and female in the case and control groups were 44.2, 41.9, 43.5, and 41.2 years, respectively. There was no significant difference between two groups in this regard (P > 0.05). Demographic characteristics are shown in Table 1. No significant difference between the two groups was observed (P > 0.05).

Household facilities such as radio, television, and refrigerator that reflect the socioeconomic status, sanitary facilities such as water source, household animal ownership (for example, horse, cow, dog) and exposure to rodents (rodents/rats in house) that are *leptospiral* exposure related data are shown in Table 2. There was a significant difference between the case and control groups in these regards (p < 0.05) except for the socioeconomic status and health center access.

Individual activities such as taking bath, supplying water from the river or brook, washing clothes and swimming in river or brook, walking barefoot or with sandals out of the house and travelling out of community were similar in both groups.

In the case group, males were at a higher risk of infection (more than two times) than females, whereas in the control group the risk of infection was the same in both genders. Farmers more than 35 years were at a higher risk of infection compared to the other age groups. These results are shown in Table 3.

**Table 1.** Demographic Characteristics of Rice Farmers and Non-rice Farmers Participating in the Leptospirosis Study

Variables	Cases (n = 144) N (%)	Controls (n = 144) N (%)	P value ^a^
Sex			
Male	86 (59.7)	78 (54.2)	0.76
Female	58 (40.3)	66 (45.8)	
**Age (years)**			
< 15	0 (0.0)	2 (1.4)	
15-35	55 (38.2)	59 (41.0)	0.52
> 35	89 (61.8)	83 (57.6)	

^a^ Statistically non- significant (P > 0.05)

**Table 2.** Household Factors of Health Facilities, Socioeconomics, Animal Contacts and Rat or Rodents in Houses Among Rice Farmers and non-Rice Farmers Participating in the Leptospirosis Study

Variables	Cases (n = 144) N (%)	Controls (n = 144) N (%)	P value
**Health center access**	140 (97.2.7)	144 (100.0)	0.12
**Safe drinking water ** ^**a**^	101 (70.1)	118 (81.9)	0.02
**Toilet ** ^**a**^	98 (68.1)	121 (84.0)	0.002
**Fair socio economic ** ^**a**^	121 (84.0)	112 (77.8)	0.23
**Animal contact ** ^**a**^	121 (84.0)	79 (54.9)	< 0.001
**Rat/rodent in house ** ^**a**^	78 (54.2)	46 (31.9)	< 0.001

^a^ Statistically significant (P < 0.05)

**Table 3.** Demographic Characteristics of IgM Anti-leptospira Antibody Positive and IgM Anti-leptospira Antibody Negative participants of the Study

Variables	Cases (n = 144)	OR (95% CI), P value	Controls (n = 144)	OR (95% CI), P value
	IgM +, IgM - N (%)		IgM + IgM - N (%)	
**Sex**		2.16 (1.05-4.48), P = 0.05		0.79 (0.22-2.28) P = 0.54
Male	37 (71.1)^a^, 49 (53.3)		6 (46.1), 72 (54.9)	
Female	15 (28.9), 43 (46.7)		7 (53.9), 59 (45.1)	
**Age (years)**		2.18 (1.04-4.56), P = 0.03		1.19 (0.37-3.85) P = 0.76
< 15	0 (0.0), 0 (0.0)		0 (0.0), 2 (1.5)	
15-35	14 (26.9), 41 (44.6)		5 (38.5), 54 (41.2)	
> 35	38 (73.1)^a^, 51 (55.4)		8 (61.5), 75 (57.3)	

^a^ Statistically significant (P < 0.05)

## 5. Discussion

In the current study, more than a third of rice farmers in the region were seropositive for leptospirosis. Rural inhabitants are mainly farmers, therefore all of them could be affected with direct contact with animals and working on farms, since most of them are mostly working in warm seasons, barefooted. Leptospirosis is called Paddy fever in Northern Iran where rice is the most frequent crop of farmers. Rice is a crop that requires a lot of water; therefore farmers have more contact with the pooled water in the farm. Most of the required water for rice farms in the rural areas is supplied from rivers or streams that are likely contaminated with the infected rodents or animals. The current study suggested that leptospirosis in the rural areas of Khuzestan engaged in rice production, as well as the Northern Iran are endemic for leptospirosis (8, 16). Previous studies from rice producing countries such as Thailand, Bangladesh, Brazil and India have documented leptospirosis as an occupational infection in the rice farms (2, 4, 15, 17).

The present study showed that farmers, in addition to the occupational environment, are also exposed to other *Leptospira* spp. contaminated sources in their place of living. Iranian farm houses, because of keeping animals such as cattle, sheep, and dog in addition to farm crops maintenance, are good places for rodent/rat entrance; therefore farmers are also directly or indirectly exposed to leptospiral infection sources at home. In the current study as well as other studies, living in villages and rural areas is associated with higher risk of contact with rodents and rats, cattle, dogs and other animals, exposure to the river and streams water with high probability of contamination with urine of rodents/rat or other animals suspected to infection with *Leptospira* species (2, 6, 11, 13, 18, 19).

Overall seroprevalence rate of leptospirosis in the region with 22.5% (ranging from 9.7% in non-farmers to 36.1% in rice farmers) indicates that leptospiral infection is a prevalent infection that should be considered. This finding, along with other reports of *leptospiral* infection suggests that leptospirosis may be a more frequent infection in the rural areas of Iran than previously described (8, 16).

*Leptospiral* infection in the current study was consistent (with a little differences) with reports from the tropical area (10, 17, 20). In a study in the Seychelles (Nicaragua), 9% of adult males had laboratory results consistent with the recent *leptospiral* infection, and 37% had evidence of past *leptospiral* infection (18). In another study conducted in Brazil, 22% of the study subjects were positive for IgM antibodies, but were asymptomatic. In a seroprevalence study of leptospirosis in Bangladesh, among serum samples from 31 individuals without a history of clinical illness and originally selected to serve as the controls, 15 (48%) were seropositive for leptospiral infection.

In that study infection among non-farmer individuals was not affected by demographic factors such as age and sex. Although more women were infected than men, the effect was not significant. The prevalence of infection among rice farmers showed significant difference between male and female. Since most of the farmers are men, many more infected men are expected. The mean age of both groups was not significantly different, but farmers older than 35 years were the most affected. Since young people are not interested to work in the villages and towns, they migrate to the cities to work. The current study results are in agreement with the results of some reports (8, 9, 16, 20), but are different from some other reports (3, 6, 17, 19). The reason for these differences is attributed to difference in the socioeconomic, life style, religious behavior, female involvement in occupational activities and geographical variations.

The current study was limited by the case identification based on a sample of cases with occupation on rice farming. Since they inhabited in the area which may be at the risk of exposure to other sources of infection, interpretation may bias with confounding factors. To compensate for the problem, control subjects of similar inhabitants but without farm occupation were matched to each case. Another limitation was serological diagnosis which may mimic the recent infection from previous infections. As mentioned in the methodology section isolation of *Leptospira* species by urine or blood culture because of long time duration and technical limitation on diagnostic capacity in the region was not done in the current study, in addition results of serological diagnosis are acceptable for the study purposes.

In conclusion, rural areas of Khuzestan especially in rice farming parts are endemic for leptospirosis. Rice farmers compared to the other residents in the rural areas are more significantly infected with leptospirosis; infection is highly affected by gender (male) and age (above 35 years). The most important source for exposure to this infection is water sources; rivers or brooks are highly suspected to be infected with *Leptospira* species. Another source of infection is farm houses where rodents and rats are frequently observed.

### 5.1. Recommendations

Since prevention is the best way to control the disease, it is advised that the farmers protect their hands and feet by rubber boots or gloves when working on the farms; travelers should avoid swimming in rivers and brooks in rural areas.

## References

[1] Adler B, de la Pena Moctezuma A (2010). Leptospira and leptospirosis.. Vet Microbiol..

[2] Tangkanakul W, Tharmaphornpil P, Plikaytis BD, Bragg S, Poonsuksombat D, Choomkasien P (2000). Risk factors associated with leptospirosis in northeastern Thailand, 1998.. Am J Trop Med Hyg..

[3] Mandell GL, Bennett JE, Dolin R (2010). Mandell, Douglas, and Bennett's principles and practice of infectious diseases..

[4] Sehgal SC (2006). Epidemiological patterns of leptospirosis.. Indian J Med Microbiol..

[5] Angnani R, Pathak AA, Mishra M (2003). Prevalence of leptospirosis in various risk groups.. Indian J Med Microbiol..

[6] Tangkanakul W, Smits HL, Jatanasen S, Ashford DA (2005). Leptospirosis: an emerging health problem in Thailand.. Southeast Asian J Trop Med Public Health..

[7] Bharti AR, Nally JE, Ricaldi JN, Matthias MA, Diaz MM, Lovett MA (2003). Leptospirosis: a zoonotic disease of global importance.. Lancet Infect Dis..

[8] Rahimi F, Vand Yousefi J, Moradi Bidhendi S, Bouzari M (2007). Leptospirosis in the rural areas of Guilan province (2004-2005).. J Kermanshah Univ Med Sci...

[9] Taghavi SAH, Nabavi M, Amini SMRR (2006). Serological study for measuring rate of leptospirosis in patients who encountered “Shaltook fever”.. Acta Medica Iranica..

[10] Ebrahimi A, Nasr Z, Kojouri GA (2004). Seroinvestigation of bovine leptospirosis in Shahrekord district, central Iran.. Iran J Vet Res..

[11] Levett PN (2003). Usefulness of serologic analysis as a predictor of the infecting serovar in patients with severe leptospirosis.. Clin Infect Dis..

[12] Levett PN, Branch SL, Whittington CU, Edwards CN, Paxton H (2001). Two methods for rapid serological diagnosis of acute leptospirosis.. Clin Diagn Lab Immunol..

[13] Sejvar J, Bancroft E, Winthrop K, Bettinger J, Bajani M, Bragg S (2003). Leptospirosis in "Eco-Challenge" athletes, Malaysian Borneo, 2000.. Emerg Infect Dis..

[14] Sehgal SC, Sugunan AP, Murhekar MV, Sharma S, Vijayachari P (2000). Randomized controlled trial of doxycycline prophylaxis against leptospirosis in an endemic area.. Int J Antimicrob Agents..

[15] Kliegman R (2011). Nelson Textbook of Pediatrics..

[16] Mansour-Ghanaei F, Sarshad A, Fallah MS, Pourhabibi A, Pourhabibi K, Yousefi-Mashhoor M (2005). Leptospirosis in Guilan, a northern province of Iran: assessment of the clinical presentation of 74 cases.. Med Sci Monit..

[17] Dias JP, Teixeira MG, Costa MC, Mendes CM, Guimaraes P, Reis MG (2007). Factors associated with Leptospira sp infection in a large urban center in northeastern Brazil.. Rev Soc Bras Med Trop..

[18] Ashford DA, Kaiser RM, Spiegel RA, Perkins BA, Weyant RS, Bragg SL (2000). Asymptomatic infection and risk factors for leptospirosis in Nicaragua.. Am J Trop Med Hyg..

[19] Sarkar U, Nascimento SF, Barbosa R, Martins R, Nuevo H, Kalofonos I (2002). Population-based case-control investigation of risk factors for leptospirosis during an urban epidemic.. Am J Trop Med Hyg..

[20] Kawaguchi L, Sengkeopraseuth B, Tsuyuoka R, Koizumi N, Akashi H, Vongphrachanh P (2008). Seroprevalence of leptospirosis and risk factor analysis in flood-prone rural areas in Lao PDR.. Am J Trop Med Hyg..

